# Editorial: Organizational culture and climate: new perspectives and challenges

**DOI:** 10.3389/fpsyg.2023.1267945

**Published:** 2023-09-15

**Authors:** Thais González-Torres, Vera Gelashvili, Juan Gabriel Martínez-Navalón, Giovanni Herrera-Enríquez

**Affiliations:** ^1^Department of Business Administration (ADO) Applied Economics II and Fundamentals of Economic Analysis, Rey Juan Carlos University, Móstoles, Spain; ^2^Business Economics Department, Rey Juan Carlos University, Móstoles, Spain; ^3^Department of Economic and Administrative Sciences, University of the Armed Forces– ESPE, Pontifical Catholic University of Ecuador, Quito, Ecuador

**Keywords:** organizational culture, organizational climate, employee behavior and attitudes, organizational behavior, organizational outcomes

Organizational culture is established in accordance with organizational aims as a set of common mental assumptions that lead to interpretation and action in firms by defining appropriate behavior for various contexts. Accordingly, it includes the values, activities, philosophy, and ideals of an organization (Martin, 2001; Rahman and Hadi, 2019).

All this is communicated and demonstrated through formal and informal behaviors as well as visual, verbal, and material artifacts representing the most tangible and visible manifestations of an organization's culture (Schein, 2010). Therefore, it could be said that organizational culture provides individuals with indications for both understanding and giving sense of what their organization is about (Ravasi and Schultz, 2006). For these reasons, organizational culture is considered a characteristic that helps firms to achieve and maintain competitive advantage and improve their bargaining power (Rahman and Hadi, 2019).

Following Moran and Volkwein (1992), organizational climate represents the shared perceptions, feelings, and attitudes that members of an organization have about its fundamental elements: the established norms, values, and attitudes of the organization's culture. This climate has the potential to influence employees' individual behavior either in a positive or negative way. While organizational culture gives the intangibles that are likely to accumulate to build the deeper psychology of people in a place, the climate offers an approach to the tangibles on which managers can focus to develop the behaviors they need for effectiveness (Chaudhary et al., 2014). Therefore, these two concepts complement each other and can be mutually useful in practice.

There is much we do not yet know about these important elements of the current business environment. Given the transversality of the notion of organizational culture and climate, the main purpose of this Research Topic is to provide a better understanding of these two concepts. To do so, we bring together a Research Topic of works addressing the role of organizational culture and climate from different perspectives. More evidence is needed to further understand how culture and climate can affect organizational outcomes or, conversely, what practices are best to improve them.

In doing so, Alonso Gallo and Gutiérrez López attempt to systematize the changes in the legislation on effective equality between men and women in business and to analyze its effect on organizational culture. The authors, therefore, delve into the adaptation of business culture to the new legal framework and the overcoming of gender stereotypes that have been guiding business management in the last decade.

On the other hand, based on the need for a contract-focused culture that emphasizes monitoring output and outcomes, Ngai et al. focus on demonstrating the effectiveness of social services through what is called evaluation capacity. To do so, the study develops the Evaluation Capacity Scale (ECS), a self-reporting instrument of NGO practitioners' capacity to conduct an effective evaluation of their service programs.

Organizational culture is often perceived as a valuable strategic asset supporting business transformation and the exploitation of digital technologies. Still, it can also be the source of inertia that impedes change. To address this issue, Busco et al. explore how digital strategy and digital leadership are the main factors affecting the acquisition of digital culture. In the same line of thought, Díaz-García et al. addressed how Higher Education Institutions (HEIs) are experiencing the cultural change resulting from digitalization and digital transformation.

Based on the idea that organizational climate and culture are instruments that lead to a better understanding of stakeholder needs, engaging them at a strategic level, and leading to a better corporate image, Álvarez-Foronda et al. try to provide an understanding of today's internal audit departments. In this sense, the authors explore how scientific literature has addressed the role of this internal function as part of the corporate governance and guardian of the organization's culture and climate, as well as the opportunities that new technologies offer to increase their effectiveness and efficiency.

Different dimensions of organizational climate are also addressed in this Research Topic. The work of Li et al. focuses on organizational results, like innovation performance. In this vein, the study explores the mechanism of team learning climate on innovation performance. Their results prove the positive effect of team learning climate on knowledge integration capability and innovation performance, among others.

The rest of the works are more focused on individual employee outcomes. Over time, numerous explanations and operationalizations have been developed for organizational climate and work satisfaction and employee wellbeing, which have been established as important pillars of research and practice in organizational behavior and organizational psychology. In this line, Santana and Pérez-Rico examine the dynamics of climate and job satisfaction in healthcare organizations from the practice and research perspectives. Accordingly, they identify measures of climate and job satisfaction used in healthcare settings, assess their psychometric properties, and appraise the overall quality of underlying studies.

On the other hand, Su and Hahn explore whether millennial employees have higher affective wellbeing in organizations with a good ethical climate. Their findings support the crucial role of a moral consensus shared by employees -ethical climate- to foster organizational citizenship behavior (OCB) and affective wellbeing of this generation of workers. Similarly, Teetzen et al. propose that organizational health climate (OHC) is an important organization-based resource for a health-oriented leadership style, which mediates the relationship between organizational health climate (OHC), employee job satisfaction, and emotional exhaustion.

Finally, and based on the idea that the organizational climate predicts organizational commitment and other employee behaviors, Gómez-Jorge and Díaz-Garrido assume that this element can also increase the level of self-esteem in the long term. In this context, the study aims to analyze the impact of self-esteem in the work environment on teaching and research productivity within the field of higher education.

Taking into account all the papers collected in this Research Topic, we strongly believe that progress has been made in the academic literature on organizational culture and climate. As organizational culture and climate is one of the topics of great interest for various stakeholders in the company, the research papers of this Research Topic can serve as a guide/guidance for improving organizational climate or culture, as these are important aspects for the proper functioning of any company in any industry.

## Author contributions

TG-T: Conceptualization, Writing—original draft, Writing—review and editing. VG: Conceptualization, Writing—original draft, Writing—review and editing. JM-N: Conceptualization, Writing—original draft, Writing—review and editing. GH-E: Conceptualization, Writing—original draft, Writing—review and editing.
